# Supplementary material for: Healthy Eating and Mortality among Breast Cancer Survivors: A Systematic Review and Meta-Analysis of Cohort Studies
Source: Int J Environ Res Public Health. 2022 Jun 21;19(13):7579. doi: 10.3390/ijerph19137579 (PMC9266181; doi:10.3390/ijerph19137579)
Supplement: Supplementary file 1 [file ijerph-19-07579-s001.zip › Table S1 Search Strategies.pdf]

Supplemental Table S1. Search Strategies.

Web of Science

Search History	Query	Results
#1	TOPIC: (breast cancer OR breast neoplasm OR breast carcinoma OR breast tumo*)<br>Indexes=SCI-EXPANDED, SSCI, A&HCI, CPCI-S, CPCI-SSH, BKCI-S, BKCI-SSH, ESCI, CCR-EXPANDED, IC Timespan=All years	**635,194**
#2	TOPIC: (diet* OR diet* quality OR diet* score OR diet* index)<br>Indexes=SCI-EXPANDED, SSCI, A&HCI, CPCI-S, CPCI-SSH, BKCI-S, BKCI-SSH, ESCI, CCR-EXPANDED, IC Timespan=All years	**889,394**
#3	TOPIC: (recur* OR relapse)<br>Indexes=SCI-EXPANDED, SSCI, A&HCI, CPCI-S, CPCI-SSH, BKCI-S, BKCI-SSH, ESCI, CCR-EXPANDED, IC Timespan=All years	**964,623**
#4	TOPIC: ((mortality or mortality rate or death or death rate) OR (survival rate or survival outcomes or clinical outcomes) OR prognosis)<br>Indexes=SCI-EXPANDED, SSCI, A&HCI, CPCI-S, CPCI-SSH, BKCI-S, BKCI-SSH, ESCI, CCR-EXPANDED, IC Timespan=All years	**3,111,197**
#5	**#1 AND #2 AND #3**<br>Indexes=SCI-EXPANDED, SSCI, A&HCI, CPCI-S, CPCI-SSH, BKCI-S, BKCI-SSH, ESCI, CCR-EXPANDED, IC Timespan=All years	**737**
#6	**#1 AND #2 AND #4**<br>Indexes=SCI-EXPANDED, SSCI, A&HCI, CPCI-S, CPCI-SSH, BKCI-S, BKCI-SSH, ESCI, CCR-EXPANDED, IC Timespan=All years	**3469**
#7	**#5 OR #6**<br>Indexes=SCI-EXPANDED, SSCI, A&HCI, CPCI-S, CPCI-SSH, BKCI-S, BKCI-SSH, ESCI, CCR-EXPANDED, IC Timespan=All years	**3828**
#8	**#5 OR #6**<br>Refined by: LANGUAGES: (ENGLISH OR SPANISH OR KOREAN)<br>Indexes=SCI-EXPANDED, SSCI, A&HCI, CPCI-S, CPCI-SSH, BKCI-S, BKCI-SSH, ESCI, CCR-EXPANDED, IC Timespan=All years	**3760**
#9	**#5 OR #6**<br>Refined by: LANGUAGES: (ENGLISH OR SPANISH OR KOREAN) AND TOPIC: (female OR women)<br>Indexes=SCI-EXPANDED, SSCI, A&HCI, CPCI-S, CPCI-SSH, BKCI-S, BKCI-SSH, ESCI, CCR-EXPANDED, IC Timespan=All years	**1899**
**#10**	**#5 OR #6**<br>Refined by: LANGUAGES: (ENGLISH OR SPANISH OR KOREAN) AND TOPIC: (female OR women) AND DOCUMENT TYPES: (ARTICLE)<br>Indexes=SCI-EXPANDED, SSCI, A&HCI, CPCI-S, CPCI-SSH, BKCI-S, BKCI-SSH, ESCI, CCR-EXPANDED, IC Timespan=All years	**1539**

Search History	Query	Results
**S1**	TOPIC: (breast cancer OR breast neoplasm OR breast carcinoma OR breast tumor Expanders – Apply equivalent subjects; Search modes – Find all my search terms	**569,3154**
**S2**	TOPIC: (diet* OR diet* quality OR diet* score OR diet* index)	**1,138,498**
**S3**	TOPIC: (recur* OR relapse)	**1,088,748**
**S4**	TOPIC: (mortality or mortality rate or death or death rate) OR (survival rate or survival outcomes or clinical outcomes) OR prognosis)	**4,555,559**
**S5**	**S1 AND S2 AND S3**	**1056**
**S6**	**S1 AND S2 AND S4**	**3881**
**S7**	**S5 OR S6**	**4339**
**S8**	**S5 OR S6**<br>Limiters – Scholarly (Peer Reviewed) Journals; Published Date: - 20210531; Human; Sex: Female; Age Related: All Adult: 19+ years; Language: English, Korean, Spanish;	**1763**

EBSCOhost (including Medline, CINAHL, and PsycINFO databases)
