# Supplementary material for: Healthy Eating and Mortality among Breast Cancer Survivors: A Systematic Review and Meta-Analysis of Cohort Studies
Source: Int J Environ Res Public Health. 2022 Jun 21;19(13):7579. doi: 10.3390/ijerph19137579 (PMC9266181; doi:10.3390/ijerph19137579)
Supplement: Supplementary file 1 [file ijerph-19-07579-s001.zip › Table S2 Quality Assessment of Included Studies.pdf]

Supplemental Table S2. Quality Assessment of Included Studies.

Author, Year	Assessment Domain									Total Score
	Selection				Comparability		Outcomes			
	A	B	C	D	E	F	G	H	I	
Kim, 2011	-	*	-	*	*	*	*	*	-	6
George, 2011	*	*	-	*	*	-	*	-	*	6
Izano, 2013	-	*	-	*	*	*	*	*	-	6
George, 2014	*	*	-	*	*	*	*	-	*	7
McCullough, 2016	*	*	-	*	*	*	*	*	-	7
Deshmukh, 2018	-	*	*	*	*	-	*	*	-	6
Sun 2018	*	*	-	*	*	*	*	*	*	8
Karavasiloglou 2019	-	*	*	*	*	*	*	*	-	7
Di Maso, 2020	*	*	-	*	*	*	*	*	*	8
Wang, 2020	*	*	-	*	*	*	*	*	*	8
Ergas 2021	*	*	-	*	*	*	*	*	*	8

A summary score was calculated using the Newcastle-Ottawa Scale for cohort studies, and studies that received a score of 6 or above were considered as high quality.

<u>Selection (maximum 4 stars):</u>

A = Representativeness of the exposed cohort

B = Selection of the non-exposed cohort

C = Ascertainment of exposure

D = Demonstration that outcome of interest was not present at start of study

<u>Comparability (maximum 2 stars):</u>

Comparability of cohorts on the basis of the design or analysis controlled for confounders

E = The study controls for age, menopausal status, and body mass index.

F = The study controls for other factors (list) including total energy intake and cancer stage.

<u>Outcomes (maximum 3 stars):</u>

G = Ascertainment of outcome

H = Was follow-up long enough for outcomes to occur

I = Adequacy of follow-up of cohorts
