# Supplementary material for: Healthy Eating and Mortality among Breast Cancer Survivors: A Systematic Review and Meta-Analysis of Cohort Studies
Source: Int J Environ Res Public Health. 2022 Jun 21;19(13):7579. doi: 10.3390/ijerph19137579 (PMC9266181; doi:10.3390/ijerph19137579)
Supplement: Supplementary file 1 [file ijerph-19-07579-s001.zip › Table S3 Excluded Studies.pdf]

Table S3. Excluded studies.

Study	Title	Reason for Exclusion
Anderson 2016	Changes in diet quality in a randomized weight loss trial in breast cancer survivors: the lifestyle, exercise, and nutrition (LEAN) study	Study examined diet quality without reporting associations with breast cancer outcomes
Farvid 2021	Postdiagnostic Dietary Glycemic Index, Glycemic Load, Dietary Insulin Index, and Insulin Load and Breast Cancer Survival	Study used other dietary index
Gold 2009	Dietary pattern influences breast cancer prognosis in women without hot flashes: the women's healthy eating and living trial	Not reporting/evaluating outcome of interest
Heidemann 2008	Dietary patterns and risk of mortality from cardiovascular disease, cancer, and all causes in a prospective cohort of women	Study population included healthy general population
Jang 2018	Association between the Dietary Inflammatory Index and Risk for Cancer Recurrence and Mortality among Patients with Breast Cancer	Study used other dietary index
Kant 2000	A prospective study of diet quality and mortality in women	Study population included healthy general population
Kroenke 2005	Dietary patterns and survival after breast cancer diagnosis	Study did not report any dietary score/index
Kroenke 2013	High- and low-fat dairy intake, recurrence, and mortality after breast cancer diagnosis.	Study did not report any dietary score/index
Krok-Schoen 2021	Prevalence of lifestyle behaviors and associations with health-related quality of life among older female cancer survivors	Study population included those with other than breast cancer or multiple cancers
Kwan 2009	Dietary patterns and breast cancer recurrence and survival among women with early-stage breast cancer	Study did not report any dietary score/index
Lopez-Pentecost 2020	Role of dietary patterns and acculturation in cancer risk and mortality among postmenopausal Hispanic women: results from the Women's Health Initiative (WHI)	Study population included healthy general population
Marchello 2020	Rural breast cancer survivors are able to maintain diet quality improvements during a weight loss maintenance intervention	Study examined diet quality without reporting associations with breast cancer outcomes
Mohammadi 2013	Association of nutritional status with quality of life in breast cancer survivors	Not reporting/evaluating outcome of interest
Palacios 2017	Dietary Patterns in Puerto Rican and Mexican-American Breast Cancer Survivors: A Pilot Study	Study examined diet quality without reporting associations with breast cancer outcomes

Pierce 2007	Influence of a diet very high in vegetables, fruit, and fiber and low in fat on prognosis following treatment for breast cancer: the Women's Healthy Eating and Living (WHEL) randomized trial	Study did not report any dietary score/index
Prieto-Ramos 1996	Mortality trends and past and current dietary factors of breast cancer in Spain	Study did not report any dietary score/index
Sasaki 1993	An ecological study of the relationship between dietary fat intake and breast cancer mortality	Study did not report any dietary score/index
Springfield 2019	Adherence to American Cancer Society and American Institute of Cancer Research dietary guidelines in overweight African American breast cancer survivors	Study examined diet quality without reporting associations with breast cancer outcomes
Thomson 2014	Nutrition and physical activity cancer prevention guidelines, cancer risk, and mortality in the women's health initiative	Study population included healthy general population
Wang 2020	Long-term anti-inflammatory diet in relation to improved breast cancer prognosis: a prospective cohort study	Study used other dietary index
Zucchetto 2017	Dietary inflammatory index before diagnosis and survival in an Italian cohort of women with breast cancer	Study used other dietary index
