# Supplementary material for: Healthy Eating and Mortality among Breast Cancer Survivors: A Systematic Review and Meta-Analysis of Cohort Studies
Source: Int J Environ Res Public Health. 2022 Jun 21;19(13):7579. doi: 10.3390/ijerph19137579 (PMC9266181; doi:10.3390/ijerph19137579)
Supplement: Supplementary file 1 [file ijerph-19-07579-s001.zip › Table S4 Quality of Evidence.pdf]

Supplemental Table S4. Summary of findings for the main comparison. High quality diet compared to low quality diet among breast cancer survivors.

Certainty Assessment							Summary of Findings				
Participants (studies) Follow-up	Risk of bias	Inconsistency	Indirectness	Imprecision	Publication bias	Overall certainty of evidence	Study event rates (%)		Relative effect (95% CI)	Anticipated absolute effects	
							With Low quality diet	With High quality diet		Risk with Low diet quality	Risk difference with High diet quality
**All-cause mortality (pre-diagnosis diet) (follow-up: range 9.8 years to 12.6 years; assessed with: National Death Index, State Vitality file)**											
4291 (3 cohort studies)	not serious	not serious	not serious	Serious [1]	none	⊕◯◯◯ Very low	641/2005 (32.0%)	639/2286 (28.0%)	**HR 0.88** (0.73 to 1.06)	320 per 1000	**32 fewer per 1000** (from 75 fewer to 16 more)
**All-cause mortality (post-diagnosis diet) (follow-up: range 6 years to 17.2 years; assessed with: National Death Index, State Vitality file)**											
9694 (10 cohort studies)	not serious	not serious	not serious	not serious	publication bias strongly suspected [2] strong association [3]	⊕⊕◯◯ Low	958.5/5117 (18.7%)	739/4577 (16.1%)	**HR 0.79** (0.70 to 0.89)	187 per 1000	**36 fewer per 1000** (from 52 fewer to 19 fewer)
**Breast cancer-specific mortality (pre-diagnosis diet) (follow-up: range 9.8 years to 12.6 years; assessed with: National Death Index, State Vitality file)**											
4291 (3 cohort studies)	not serious	not serious	not serious	not serious	none	⊕⊕◯◯ Low	259/2005 (12.9%)	280/2286 (12.2%)	**HR 0.97** (0.81 to 1.17)	129 per 1000	**4 fewer per 1000** (from 23 fewer to 20 more)
**Breast cancer-specific mortality (post-diagnosis diet) (follow-up: range 6 years to 17.2 years; assessed with: National Death Index, State Vitality file)**											
11334 (9 cohort studies)	not serious	Serious [4]	not serious	Serious [1]	publication bias strongly suspected [2]	⊕◯◯◯ Very low	530.25/5937 (8.9%)	446/5397 (8.3%)	**HR 0.85** (0.62 to 1.18)	89 per 1000	**13 fewer per 1000** (from 33 fewer to 15 more)

CI: confidence interval; **HR:** hazard Ratio

Explanations

1. Downgraded due to 95% CI includes both null value and appreciable benefit or appreciable harm (i.e., HR of under 0.8 or over 1.2), which suggests the risk of very serious imprecision of the results and thus low confidence in their reliability
2. Downgraded due to publication bias as funnel plot indicates missing publications from a small study with null/negative results.
3. Upgraded due to a large effect (i.e., HR of under 0.8 or over 1.2), which suggests the importance of the outcomes and thus high confidence in their estimate
4. Downgraded due to heterogeneity I2=61.7% and p <0.05, which suggests a serious risk of inconsistency (unexplained heterogeneity), which may arise from relevant differences in populations, exposure, and outcomes of the studies entered into the analysis
